# What are the key challenges we face in kidney transplantation today?

**DOI:** 10.1186/2047-1440-2-S1-S1

**Published:** 2013-11-20

**Authors:** Jeremy R Chapman

**Affiliations:** 1 Centre for Transplant and Renal Research, University of Sydney, Westmead Hospital, Westmead, NSW 2145, Australia

## Abstract

Transplantation is more predictable than it was 20 to 30 years ago and innovation over the last 20 years has been rapid, delivering substantial short-term and medium-term improvements. The challenges ahead are to deliver improved results globally in the context of also preventing chronic disease and reducing the costs of treatment. Countries achieving the best rates of transplantation combine deceased and living donors and can transplant more than 50 people per annum per million population, so why can this not be achieved everywhere? The mortality rates have dropped, but they are still up to 10-fold worse than age- and sex-matched controls, such that transplantation ages individuals by 30 years in terms of mortality risk. Cardiovascular disease, infection and malignancy remain the targets if mortality is to normalize. Graft survival rates will not change until the multiple injuries constituting chronic allograft dysfunction and the problems of recurrent disease can be brought to heel. Biomarkers may provide the next innovation to advance outcomes, but early experimental tolerance protocols implemented in clinical practice in at least three centers may deliver results more quickly.

## Introduction

Transplantation today is a far cry from the field that encouraged many current practicing clinicians to take this career path. The results are much more predictable than they were 20 to 30 years ago and the investigative and therapeutic tools we have at our disposal are much more powerful. Some of the diseases we used to treat are rare or have vanished, such as analgesic nephropathy, to be replaced by a depressing avalanche of diabetic and hypertensive nephropathy in increasingly older patients. The pace of innovation over the last 20 years has been rapid and we have become used to seeing continuous and substantial improvements, but there is the concern that the field is stagnating, partly because those innovations have brought results that seem hard to improve upon. The excitement of innovation may have passed to another field – perhaps oncology, perhaps intraluminal intervention – and we are left with the feeling in transplantation that we can only tidy up our results at the margins. In this paper I will review whether or not this situation is true and consider some of the challenges that are either with us or ahead of us.

## The incidence and prevalence of treated and untreated end-stage kidney disease

### Incidence of chronic kidney disease

How many people develop end-stage kidney disease (ESKD) remains a perennial question for clinicians, managers and health policy analysts as well as the treasuries that fund treatment. The answer is hard to find since the untreated patients die and are not to be found in the hospital statistics or in registries of dialysis or transplantation patients. The patients who die untreated may not be seen by specialist physicians or may never be admitted to a hospital; they may in fact never be diagnosed or ever be seen by a doctor in many countries. In advanced western economies, however, death certificate records are one way of assessing the causes of death of the population, and while they have their weaknesses, these records can provide reasonable estimates of need.

**Figure 1:**
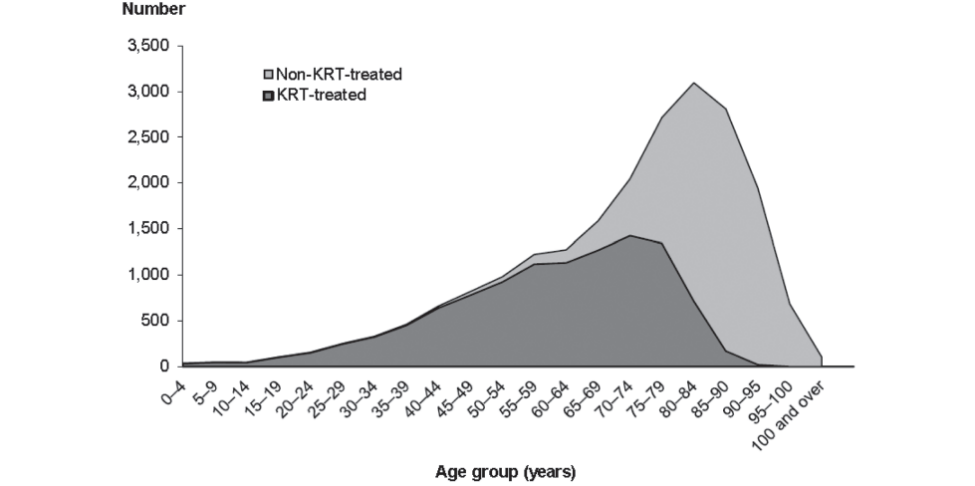
**Comparison of treated and untreated end-stage kidney disease in Australia between 2003 and 2007.** KRT, kidney replacement therapy. Reproduced with permission from [1].

The Australian Institute of Health and Welfare has compiled death records of patients identified as having died primarily of chronic kidney disease (CKD) and correlated them with the records of the Australian and New Zealand Dialysis and Transplant Registry to determine which patients had been treated and which had not been treated by dialysis or transplantation [1]. The resultant analysis demonstrated that most Australians under the age of 60 years had been treated by dialysis or transplantation, while most over 80 years old had not (Figure 1). That this is country specific is clarified by the fact that the maximal combined incidence of both treated and untreated ESKD in Australia is lower than the United States incidence of treated ESKD. This fact and the great variation of incidence by population – for example, the Aboriginal population in Australia has extremely high rates – highlights the need for a focus on prevention of CKD through active public health and therapeutic interventions. The past 5 years have, in Australia, seen a stabilizing of incidence of new dialysis patients younger than 75 years and now for 3 years a progressive decrease in new patients. No consideration of renal transplantation can thus ignore the relative investment needed in prevention of CKD, especially in the emerging and developing economies of the world, and the Australian experience suggests that this is a legitimate and realistic target.

The first Key Challenge is thus to prevent CKD and retard progression to ESKD.

### Incidence of transplantation

**Figure 2:**
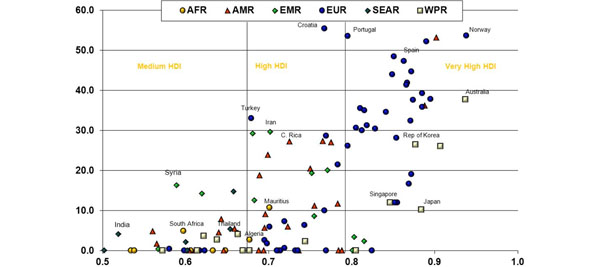
**Total number of kidney transplants per million population**. Total number of kidney transplants per million population correlated against the Human Development Index (HDI) for member states of the World Health Organisation (WHO). AFR, WHO African Region; AMR, WHO Region of the Americas; EMR, WHO Eastern Mediterranean Region; EUR, WHO European Region; SEAR, WHO South-East Asia Region; WPR, WHO Western Pacific Region. Reproduced with permission from [2].

The incidence of ESKD that is treated by renal transplantation varies around the world, as can be seen in Figure 2 that is derived from the World Health Organisation Global Observatory on Donation and Transplantation [2]. The highest rates of transplantation are thus seen in Croatia, Norway and Portugal through a combination of donation by both deceased and living donors, while Spain has the highest rate of deceased organ donation of any of the more populous nations. From these data one can derive a benchmark rate of over 50 kidney transplants per million population per annum. The vast majority of even the most developed countries with the highest Human Development Index scores are thus failing to reach this benchmark rate, which has been demonstrated to be possible. There were approximately 75,000 kidney transplants globally in 2010, but this benchmark rate would call for 350,000 transplants, or four to five times the rate achieved today.

The second Key Challenge is thus to raise the rate of kidney transplantation to 50 or more transplants per annum per million population in all countries.

### Costs of treatment

While there are many difficulties in estimating the cost of treating kidney disease, this has been estimated by many policy analysts, by industry and by lobby groups in many countries. None of the estimates or projections is affordable. Dialysis and transplantation are expensive therapies and the cumulative impact of survival leads to escalating total costs that are straining the US Medicare budget and in most countries see a disproportionately high percentage of healthcare resources disbursed to a small sector of the population. Projections in Australia are that the total cost of treatment for ESKD will be between AUS $11.3 billion and AUS $12.3 billion by 2020, having risen from AUS $1 billion in 2009 [3].

The third Key Challenge is thus to reduce the total costs of treatment for ESKD by optimizing the rate of kidney transplantation and reducing the cost of dialysis and the cost per transplant.

## Outcomes of transplantation

### Patient survival

The most important outcome of transplantation from the patient’s perspective is their survival even in the event of failure of the graft. This has become a more certain outcome in the past decade but is still far from acceptable. The mortality rate for transplant recipients in the 1970s was as high as 40% in the first year and so we now congratulate ourselves on a mortality rate that in developed countries is approximately 3% at 1 year [4]. Comparison with the general population and the dialysis population demonstrates the superiority of transplant over dialysis, but also demonstrates a wide discrepancy between the transplant population and the general community [4]. In fact, the survival of transplant recipients by age cohort is more akin to the survival of the general population with a diagnosis of cancer [5]. Depending upon the age of the patient, the excess risk of death is up to 10-fold the normal population; or to put it another way, the risk of death for a transplant recipient is equivalent to that of an average person 30 years older. Perhaps there is not so much to congratulate ourselves on as there is to challenge ourselves with?

### Causes of death

The causes of death after transplantation have been changing slowly over the past decades and also vary with the time after transplantation. Infection, cardiovascular disease and malignancy are the three most important causes of death. The death rates from cardiovascular disease in the Australian transplant population has decreased over the past 20 years from 1.8 deaths per 100 patient-years in the mid-1980s to 0.9 deaths per 100 patient-years in the mid-2000s, while infection has decreased from 0.7 to 0.3 deaths per 100 patient-years. Malignancy shows the reverse, with a slight increase from 0.6 to 0.8 deaths per 100 patient-years, and has almost become the commonest cause of death, surpassing combined cardiovascular diseases. If we are to impact beneficially on patient mortality there is more to be done with respect to all three main causes, but of the three we have the least armamentarium against cancer.

The fourth Key Challenge is to reduce the mortality rate after kidney transplantation through improving cardiovascular health, reducing infection risks, and detecting and treating cancer.

### Graft survival

**Figure 3:**
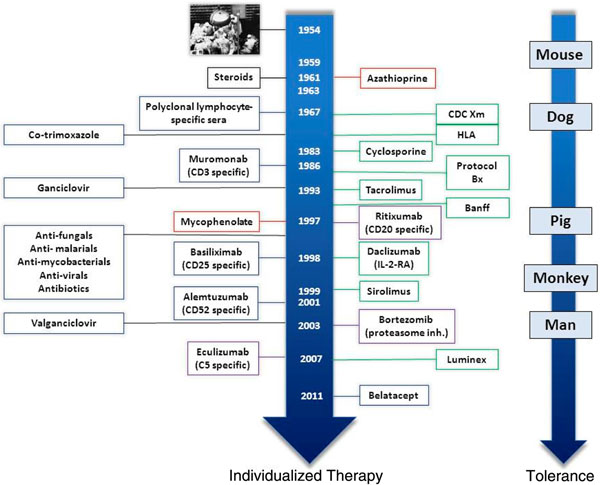
Development of therapeutic agents in transplantation.

The live patient’s priority is clearly to have a functioning kidney transplant through avoiding rejection. This has been accomplished to a great degree in the first year after transplantation, with graft loss now quite rare from rejection. By weighting both sides of the scales we have achieved a remarkable reduction in acute rejection rates and also in graft loss from acute rejection. We have increased immunosuppressive potency but at the same time we have – at least in the affluent world – also increased the use of prophylactic agents for the infectious agents against which we have effective strategies – cytomegalovirus and *Pneumocystis jurovecii* (Figure 3). This strategy has left our grafts at the mercy of infectious agents that we cannot today control, such as BK virus, and funguses that regularly take a toll in both the developed and emerging economies of the world.

**Figure 4:**
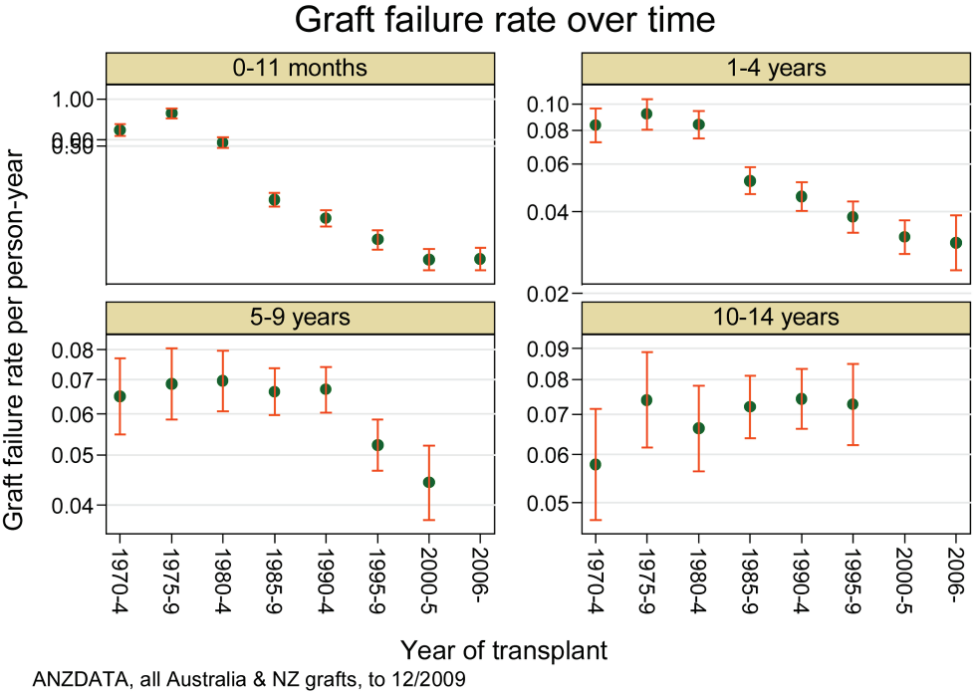
**Graft survival in Australia and New Zealand from the 1960s to today.** Short-term, medium-term and long-term graft survival in Australia and New Zealand from the 1960s to today. Reproduced with permission from [4].

The well-known Australian and New Zealand Dialysis and Transplant Registry analysis of long-term outcomes [5] demonstrates quite nicely the impact we have had on graft loss in the first year (Figure 4, top-left panel), contrasted with the relative failure to impact graft survival beyond 10 years (Figure 4, bottom-right panel). We have made a large impact on short-term outcomes and this is our success, but we have hidden our failure to impact the long term because it is hard to follow enough patients for long enough to even understand the crude outcomes, let alone visualize and investigate the causes of differences in long-term results.

### Causes of graft loss

Death with a functioning graft accounts for about one-half of the graft losses in most series, with the remainder being attributable either to a miscellany of issues such as recurrence of glomerulonephritis, vascular thrombosis, technical complications and a few acute rejection losses, or to the constellation of causes of chronic progressive dysfunction of the graft. Variably named over the years as chronic rejection, chronic allograft nephropathy and now chronic allograft dysfunction, this condition is seen as a mystery by some and explicable by others [6].

The kidney has limited responses to injury and thus anything that exerts a negative impact on the kidney tends to lead to fibrosis and atrophy of the tubules with glomerulosclerosis. A common misconception is that a particular kidney can only be affected by one problem at a time. The injuries that contribute to chronic dysfunction start in the donor, and relate to the actual transplant procedure through the degree of ischemia; to the bouts of acute clinical and subclinical rejection that can occur in the early weeks (especially if untreated); as well as the nephrotoxicity of the calcineurin inhibitors we use to protect the kidney from rejection; and also the insidious damage resulting from chronic antibody-mediated rejection. There are no biological laws that restrict damage to only one of those entities.

The fifth Key Challenge is to reduce the rate of graft loss through focus on subclinical phenomena, understanding the role of antibodies to donor HLA antigens and improving the way in which we use nephrotoxic drugs to retain their short-term advantages but ameliorate their long-term disadvantages.

## Innovation in transplantation

The optimist will look at what we have achieved in the clinic over the past 40 years and identify the truth of the impact of sometimes serendipitous innovation. These same people will look forward to innovation to provide the next wave of improvements in our outcomes. There are many that say the days of relying on serendipity are behind us, so perhaps it will be through the science of today that we will see the clinical advances of tomorrow. If so, then I would nominate biomarkers and clinical tolerance strategies as the most likely contenders for the crown.

### Biomarkers

The first and most successful biomarker in renal transplantation was serum creatinine, and it would be fair to suggest that we owe the success of renal transplantation to measuring this molecule reliably and at low cost to predict when increased immunosuppression is needed to combat acute rejection. There is a substantial stream of research directed at identifying new molecules that can, in urine or blood, predict both short-term and long-term outcomes such as acute allograft rejection and graft fibrosis [7]. There is also a clear line of research that is aimed at using biomarkers to identify the cellular mechanisms involved in development of spontaneous tolerance.

The promise of biomarkers in other fields, such as cancer therapy, is that the combination of a biomarker and a specific therapeutic agent will advance the results for particular patients. Can this strategy lead to the best use of immunosuppression where the increased visibility of humoral rejection mechanisms has directed our attention to anti-B cell-strategies, yet the precise B-cell profile seems to be relevant to identifying spontaneously tolerant individuals? Can biomarkers solve our conundrums around HLA-sensitized recipients? The fundamental uncertainty over what is and what is not patentable may be behind us with recent pivotal decisions in the United States – fundamental laws of nature biology and genetics are not patentable, but the tests that measure them and that are applicable in a nonobvious manner to modify therapy are patentable.

The sixth Key Challenge is to use biomarkers to better guide and individualize therapy.

### Tolerance

Medawar and his colleagues in the 1950s conceived and demonstrated that allogeneic organ transplantation did not need immunosuppression through strategies designed to either evade the alloimmune response or suppress, delete or regulate it. Three clinical programs in Boston, Stanford and Chicago have shown that this may be possible in man, in which a total of about 40 patients have so far been reported [8-10].

The strategy for creating mixed allogeneic chimerism has been achieved in slightly different ways in the three programs. In Boston, simultaneous full bone marrow and renal transplantation from the same donor was successful in six patients with myeloma and HLA-identical siblings. This was followed with haplo-identical donors augmented by T cell depletion by antibodies and thymic irradiation. The Stanford group has undertaken 16 HLA-matched donor transplants with total lymphoid irradiation, anti-thymocyte globulin, and donor CD34^+^ hematopoietic progenitor cells and T cells. Louisville and Chicago have reported eight patients treated with total body irradiation, nonmyeloablative chemotherapy and an HLA-mismatched renal transplant from a living donor, followed on day +1 with infusion of tolerance-promoting facilitator cells and mobilized hematopoietic stem cells. Five of these eight patients have been weaned off immunosuppression.

These clinical outcomes are of course exciting but there is much work to be undertaken before this approach can be seen as an acceptable alternative to conventional transplantation. Perhaps the bar has been set too high and what we should seek instead is definition of those patients for whom a stable state may be achieved with low levels of immunosuppression. We must be mindful of the patients unlikely to be managed through tolerance induction strategies, including those sensitized with HLA antibodies and perhaps those with a risk of recurrent glomerulonephritis, and we are as yet uncertain of the long-term outcomes.

The seventh Key Challenge is formally to examine an optimized tolerance protocol in an extensive clinical trial.

## Conclusions

Despite excellent results from renal transplantation there are several key challenges today, reordered to suggest the relative importance and chance of reaching these goals:

♦ To raise the rate of kidney transplantation to 50 or more transplants per annum per million population in all countries.

♦ To reduce the mortality rate after kidney transplantation through improving cardiovascular health, reducing infection risks and detecting and treating cancer.

♦ To reduce the rate of graft loss through focus on subclinical phenomena, understanding the role of antibodies to donor HLA antigens and improving the way in which we use nephrotoxic drugs to retain their short-term advantages but ameliorate their long-term disadvantages.

♦ To prevent CKD and retard progression to ESKD.

♦ To reduce the total costs of treatment for ESKD.

♦ To use biomarkers to better guide and individualize therapy.

♦ To examine an optimized tolerance protocol in an extensive clinical trial.

There is indeed work still ahead for a generation of research scientists and clinicians.

## Abbreviations

CKD: chronic kidney disease; ESKD: end-stage kidney disease; HLA: human leukocyte antigen.

## Declarations

JRC was provided with travel expenses and an honorarium to attend and present this material and an honorarium for writing the paper.

This article has been published as part of *Transplantation Research* Volume 2 Suppl 1, 2013: Proceedings of the 12th International Symposium on Transplantation. The full contents of the supplement are available at http://www.transplantationresearch.com/supplements/2/S1. The supplement is based on the 12th International Transplantation Symposium held on 16–17 April 2012, in Istanbul, Turkey. The symposium and publication based on these proceedings were supported by an unrestricted educational grant from Pfizer Inc., who had no influence on the editorial content. Editorial support was provided by Synergy, Richmond, UK and was funded by Pfizer Inc. Rapamune (sirolimus) is a product marketed by Pfizer Inc. The articles have been through the journal’s standard peer review process and the Supplement Editors declare that they have no competing interests.
